# Better Late Than Never: Effects of Late ACA Medicaid Expansions for Parents on Family Health-Related Financial Well-Being

**DOI:** 10.1177/00469580221133215

**Published:** 2022-11-10

**Authors:** Caitlin McPherran Lombardi, Lindsey Rose Bullinger, Maithreyi Gopalan

**Affiliations:** 1 University of Connecticut, Storrs, CO, USA; 2 Georgia Tech, Atlanta, GA, USA; 3 Pennsylvania State University, University Park, PA, USA

**Keywords:** low-income, children, health insurance, Affordable Care Act, Medicaid, financial well-being, medical expenses, job lock

## Abstract

Public health insurance eligibility for low-income adults has improved adult economic well-being. But whether parental public health insurance eligibility has spillover effects on children’s health insurance coverage and family health-related financial well-being is less understood. We use the 2016 to 2020 National Survey of Children’s Health (NSCH) to estimate the effects of Medicaid expansions through the Affordable Care Act (ACA) for parents on child health insurance coverage, parents’ employment decisions due to child health, and family health-related financial well-being. We compare children in low-income families in states that expanded Medicaid for parents after 2015 to states that never expanded in a difference-in-differences framework. We find that these expansions were associated with increases in children’s public health insurance coverage by 5.5 percentage points and reductions in private coverage by 5 percentage points. We additionally find that parents were less likely to avoid changing jobs for health insurance reasons and children’s medical expenses were less likely to exceed $1000. We find no evidence that the expansions affected children’s dual coverage and uninsurance. Our estimates are robust to falsification and sensitivity analyzes. Our findings also suggest that benefits on children’s medical expenses are concentrated in the families with the greatest financial need.

**What do we already know about this topic?**
Public health insurance eligibility for low-income adults has improved adult economic well-being.
How does your research contribute to the field?
We examine if parental public health insurance eligibility has spillover effects on children’s health insurance coverage and family health-related financial well-being.
What are your research’s implications toward theory, practice, or policy?
Our study demonstrates the benefits of public health insurance expansions for families with children and suggests that the benefits of lower children’s medical expenses may be concentrated in the families with the greatest financial need.

## Introduction

Prior to the Affordable Care Act (ACA) Medicaid expansions, 36% of families experienced health-related financial burdens, such as problems paying medical bills.^1^ Health-related medical expenses comprise a substantial portion of a household’s expenses and can pose a significant financial burden on families,^2^ particularly those with children and low-incomes.^1,3^ Health-related burdens can also restrict adults’ ability to change jobs due to preexisting conditions or need to maintain employer sponsored health insurance coverage, a phenomenon known as job lock.^4,5^

The Medicaid program has long offered public health insurance coverage to very low-income adults. Before the ACA, affordable health insurance coverage options were limited for low-income families ineligible for Medicaid. The ACA provided federal funds to states to expand existing Medicaid eligibility to nonelderly, non-disabled adults with incomes below 138% of the federal poverty line (FPL). Additionally, the ACA marketplaces enabled uninsured adults without employer sponsored insurance to purchase insurance with income-related subsidies. The effect of these Medicaid expansions has been studied extensively, with evidence that they improved adults’ healthcare coverage and access, finances, and health.^6-9^ Specifically, the expansions reduced healthcare related strain,^10^ out-of-pocket expenses for medical care,^11-13^ medical bills,^14^ and the probability of bills going to collections.^15^ The expansions also reduced unpaid bills and general debt, prevented delinquencies, and improved credit scores,^16,17^ and reduced the use of payday loans^15,18^ and fringe banks.^19^ The expansions also improved housing and food security.^20-22^

The existing literature has primarily focused on “childless adults” (i.e., those without dependent children). However, the ACA Medicaid expansions also changed eligibility thresholds for low-income parents and is credited with reducing uninsurance rates among parents from 18% in 2013 to 11% in 2017.^23^ The changes to the Medicaid program for parents varied across states due to different state-level income thresholds prior to the ACA. Among the 3 expansion states examined in this study, the income eligibility limits for working parents for Medicaid pre-ACA as percent of the FPL were: 24% in Louisiana, 52% in Virginia, and 105% in Maine. Following the ACA Medicaid expansions, the income eligibility limit increased to 138% FPL in each of these states.

The goal of this paper is to determine whether these expansions in Medicaid eligibility for parents impacted their children’s public health insurance coverage and their families’ health-related employment decisions and financial well-being. We contribute to the existing literature by (1) focusing specifically on parents, (2) examining employment decisions specifically due to child health reasons and child-specific medical expenditures, and (3) providing evidence on late Medicaid expansion states. We use child-level data from the National Survey of Child Health (NSCH)^47^ to compare Medicaid-eligible households in 3 late expansion states (i.e., those that expanded between 2016 and 2019) to those in non-expansion states.^a^ The strength of the NSCH is that it provides children’s health insurance coverage status, precise income measures, and detailed measures of families’ healthcare costs and decision making. We examine contextual measures of health-related financial well-being, including parent employment changes due to health issues or insurance (i.e., job lock) and high child medical costs.

Consistent with previous research,^24^ we find that the Medicaid expansions for low-income parents were associated with increases child public health insurance coverage. Our estimate is remarkably similar to previous research, documenting increases of 5.6 percentage points (or about 8.5%) compared to Hudson and Moriya’s^24^ estimate of 5.7 percentage points (8.1%). In contrast to their findings of no significant change in private coverage, however, we find evidence of crowding out private insurance; children’s private insurance coverage declined by 4.9 percentage points (30%).

As a new contribution to the literature, we also find a reduction of about 4.5 percentage points (roughly 55%) in the likelihood that parents avoid changing jobs for reasons related to health insurance. Finally, we find the likelihood of a child’s out-of-pocket medical expenses exceeding $1000 is 4 percentage points lower, a 70% decline, suggesting the benefits of children’s health-related financial expenses are concentrated in the highest medical expense population. We do not find significant effects of the ACA Medicaid expansions on a child having both private and public insurance (dually covered) or being uninsured. Taken together, these findings demonstrate the spillover effects of the Medicaid expansions to children’s health insurance coverage, parents’ employment, and family financial well-being.

## Background

A primary goal of health insurance is to smooth the financial risk associated with poor health outcomes. There are several potential mechanisms through which the ACA Medicaid expansions may influence health-related financial well-being among families. First, expanding public health insurance programs to parents has been shown to increase health insurance coverage of both parents^25,26^ and children through “welcome mat” effects.^24,25^ Health insurance rates among children have steadily increased over the past several decades in response to state expansions of coverage for children through Medicaid and the Children’s Health Insurance Program (CHIP).^27^ In 2016, at the start of this study, 95.5% of children in the U.S. had public or private health insurance.^28^ Yet, research has shown that the Medicaid expansions increased public health insurance coverage for children who were already eligible for coverage, but not yet enrolled.^24^ Public health insurance expansions may also crowd out private insurance coverage due to Medicaid’s substantially lower premiums and copays than private insurance, which may improve the financial well-being of low-income families.

Research has documented the financial benefits that come with health insurance coverage.^10,14^ Indeed, the ACA expansions have reduced financial burdens of healthcare costs among families with children.^26,29,30^ For example, Wisk and colleagues^30^ found that low- and middle-income families who were eligible for Medicaid and insurance subsidies through the ACA Medicaid expansions experienced greater reductions in healthcare–related financial burden after the ACA was implemented compared to families with higher incomes. They were unable to account for state-specific ACA Medicaid expansion policies, however. Similarly, McMorrow and Kenney and colleagues^26,29^ found that low-income parents reported fewer problems in paying family medical bills due to expanded Medicaid coverage.

Second, the documented improvements in health among beneficiaries of the ACA Medicaid expansions may indirectly have implications for financial stability. For example, the expansions have been linked to adults’ physical health,^14,31^ including parents,^26,32^ and behavioral health.^33-36^ Bullinger^37^ also finds improvements in child support payments. Together, this literature implies that the expansions created more financial security and stability among low-income parents.

Third, the ACA Medicaid expansions may have benefited adults by reducing job lock.^4,5^ With a few exceptions,^38,39^ research on adults (not parents specifically), however, has largely found that the ACA expansions did not result in changes in employment, job switching, or reduced work hours.^38,40-43^ Previous Medicaid expansions for parents during the 1990s have been linked with reductions in job,^44^ though no known research has examined this question among parents in relation to the ACA expansions. The distinction between adults and parents may be important since parents have unique motivations for seeking certain types of employment and schedules due to caretaking responsibilities.^45,46^ Understanding whether parents are more sensitive to changes in health insurance eligibility because of the ACA in comparison to prior expansions (e.g., Hamersma and Kim^44^) is important for policy.

Taken together, evidence shows that the ACA Medicaid expansions had positive benefits for adults’ financial well-being,^6,7^ however less research exists explicitly on adults with dependent children. Among these parents, there are similar and unique mechanisms through which the expansions may have impacted health-related financial well-being, including changes in child health insurance coverage, child health related costs, and employment changes. We contribute to the existing literature by examining the effects of parental public health insurance eligibility on child health insurance coverage, parents’ employment decisions due to child health reasons, and family health-related financial well-being.

## Methods

### Data

We use the 2016 to 2020 waves of the National Survey of Children’s Health (NSCH).^47^ The NSCH is a nationally representative survey of households with children aged 0 to 17 directed by the Health Resources and Services Administration’s Maternal and Child Health Bureau.^47^ The repeated, cross-sectional survey includes robust information on children’s health and well-being including health insurance coverage, healthcare access, and medical costs, as well as household income and state of residence. We pair child-level data from the NSCH with state-level Medicaid policies from Kaiser Family Foundation^48^ to determine parental eligibility for Medicaid through the ACA.

### Sample

Due to the available data in the NSCH, we first limit the analytic sample to children residing in states that expanded between 2016 and 2019 and states that had not expanded by 2020. We exclude children in states that expanded prior to 2016 to avoid potential dynamic effects of the expansions from those earlier expanding states.^b^

**Figure 1.**
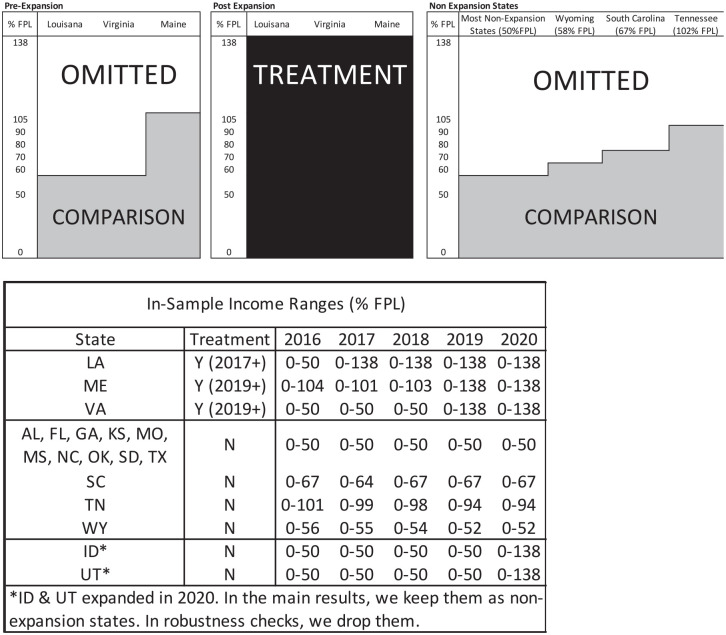
Conceptual Map of Treatment and Comparison Groups. ***Note*:** The NSCH bottom codes income as a percent of FPL at 50. Because some of these households would be considered newly eligible in expansion states with pre-ACA parental eligibility thresholds less than 50% FPL, and there is evidence of a welcome mat effect among the previously eligible (eg, McMorrow and Kenney & Kenney^29^) we consider all households with incomes <50% in expansion states to be in the treatment group. Similarly, although some households with incomes <50% FPL in non-expansion states were not eligible, they are coded as the comparison group since we cannot distinguish between them and those who were eligible.

The parental eligibility thresholds for Medicaid differed across states prior to ACA, as well. Therefore, we next limit the sample to children in households with incomes below their time-variant, state-specific eligibility thresholds to precisely capture children in households who would have been newly affected by the ACA Medicaid expansions.^c^ For example, in Virginia, parents whose household income was less than 38% of the FPL were eligible for Medicaid prior to the ACA expansions. In Maine, the threshold was much higher pre-expansion: 105%. Upon expanding their Medicaid programs through the ACA, all adults whose household income was less than 138% of the FPL became eligible. Most non-expansion states had eligibility thresholds below 50% FPL. Therefore, before the expansion, households with incomes below these thresholds are in the sample, while after expansion households with incomes below 138% FPL are in the sample in expansion states. In non-expansion states, the sample consists of households with incomes 102% FPL or less (depending on the state and year, see Figure 1). In Supplemental analyses, we test the sensitivity of the results to alternative samples.

### Measures

We focus on 2 sets of outcomes in this study: (1) health insurance coverage for children and (2) family health-related financial wellbeing due to children’s healthcare use. All measures are at the child-year level from the NSCH data.

#### Health insurance coverage

In each year of the survey, parents are asked how the child is currently insured. We code this measure as 4 mutually exclusive binary indicators (publicly; privately; both publicly and privately; or not insured) equaling 1 if the parent responded yes (Yes = 1; *0* = No).^d^

#### Health-related financial wellbeing outcomes

We use 2 measures to assess family’s health-related, financial outcomes. Each year of the survey, parents reported (a) whether they or other family members avoided changing jobs because of concerns about maintaining health insurance for the focal child in the last 12 months (Yes = 1, No = 0); and (b) their out-of-pocket medical costs for the child’s medical, health, dental, and vision care in the past 12 months in 5 categories (1 = $0, 2 = $1-$249; 3 = $250-$499, 4 = $500-$999, 5 = $1000-$5000, 6 = More than $5000).^e^ Following previous literature on the topic,^49-52^ we recoded this variable to a binary indicator (1 = $1000 or more; 0 = Less than $1000).^f^

We also adjust for several child- and family-level covariates (sex, race/ethnicity, and others) guided by past literature linking children and parents’ health insurance status.^53^ We also adjust for several state-level time-varying characteristics (e.g., unemployment rates, Earned Income Tax Credit rates, and others) that may differentially affect the outcome measures across states (see Table 1 and Appendix A for a description of control variables).

**Table 1.** Descriptive Statistics.

	Expansion state (Louisiana, Maine, Virginia)		Non-expansion states	
	Pre-expansion	Post-expansion	2016-2018	2019-2020
Outcomes				
Child publicly insured	0.64	0.64	0.63	0.55
Child privately insured	0.21	0.19	0.20	0.27
Child publicly and privately insured	0.06	0.07	0.05	0.06
Child uninsured	0.09	0.10	0.11	0.12
Avoided Changing jobs due to concerns about maintaining health insurance for child	0.08	0.05	0.04	0.06
Child medical costs exceed $1000	0.06	0.05	0.06	0.08
Household/Child variables				
Household Speaks English (Primary language)	0.92	0.92	0.87	0.88
Non-Hispanic White	0.73	0.57	0.59	0.61
Non-Hispanic Black	0.15	0.32	0.25	0.26
Other race	0.13	0.11	0.16	0.13
Hispanic	0.06	0.09	0.18	0.18
Mother is married	0.44	0.39	0.37	0.41
Mother education ≤ High School	0.07	0.09	0.13	0.11
Mother education = High School Grad	0.29	0.30	0.28	0.27
Mother education ≥ High School	0.49	0.44	0.42	0.43
Mother employed	0.36	0.47	0.37	0.45
Three or More Children in Household	0.31	0.27	0.29	0.31
Child is male	0.50	0.50	0.52	0.53
Child’s age (years)	8.7	9.3	8.8	9.3
Child is under 6 months	0.01	0.01	0.02	0.01
Household income (% FPL)	61.48	80.51	52.26	56.99
State variables				
Unemployment rate	0.04	0.05	0.04	0.05
State minimum wage ($)	8.03	8.16	7.39	7.40
State EITC rate	0.09	0.08	0.02	0.05
EITC refundable	0.33	0.81	0.09	0.04
Maximum TANF/SNAP benefit for family of 3 ($)	955	860	808	831
N	285	940	1680	1477

*Source*. NSCH 2016 to 2020.

*Note*. Sample consists of households with incomes at or below their state’s parental eligibility thresholds. Descriptive statistics are not weighted due to limits on the analytic sample.

### Statistical Analysis

Originally, the ACA required all states to expand Medicaid to all adults below 138% of the FPL. However, in 2012, the Supreme Court allowed states to opt out of this requirement. As a result, to date, 39 states including Washington, D.C. have expanded Medicaid, a largely political decision.^54^ Most states expanded in 2014, though some expanded later. We restrict our analysis to states that expanded in 2016 or later and those that did not expand at all due to lack of comparable NSCH data prior to 2016. See Appendix B for the timing and expansion status for these states.

We use this variation in the state-level ACA Medicaid expansions between 2016 and 2020 to identify the effect of parental Medicaid eligibility on children’s health insurance coverage and parents’ health-related financial wellbeing. We compare changes in outcomes between eligible parents residing in states that expanded to eligible parents in states that did not expand using a difference-in-differences (DD) approach. Therefore, we compare eligible parents in the expansion states to always eligible parents in the comparison group. See Figure 1 for a conceptual description of the treatment and comparison groups.^g^

Specifically, we estimate the following baseline linear probability model using OLS estimation^h^:

(1) $$\begin{array}{l} Y_{isy}=\beta_{0}+\beta_{1}Expansion_{sy}+\;\delta^{\prime }\;X_{i}\\ \;\;\;\;\;\;\;\;\;+\alpha Z_{sy}+\gamma_{s}+\tau_{y}+\epsilon_{isy} \end{array}$$

Where *Y_isy_* is the outcome for child *i*, living in state *s*, during year *y*. Expansion_sy_ = 1 if child i lives in one of the 3 states that expanded between 2016 and 2019 (Louisiana, Virginia, and Maine), and 0 if the child resides in a non-expansion state. For non-expansion states, the pre-ACA parental income eligibility threshold is also the contemporaneous threshold. $\gamma_{s}$ and $\tau_{y}$ are state and year fixed effects respectively, *X_i_* refers to a vector of child- and family-level characteristics, and *Z_sy_* refers to time-varying state-level characteristics.

Because the analytic sample is made up of only 18 states, we cannot rely on standard clustering of the standard errors at the state level.^55^ The standard errors will be artificially low, leading to over-rejection of the null hypothesis of no effect, thereby finding an effect when there is none. Instead, we implement a wild cluster bootstrap with 1000 replications, which provides more reliable standard errors for inference when there are too few clusters.^56-60^

## Results

### Descriptive Statistics

**Figure 2.**
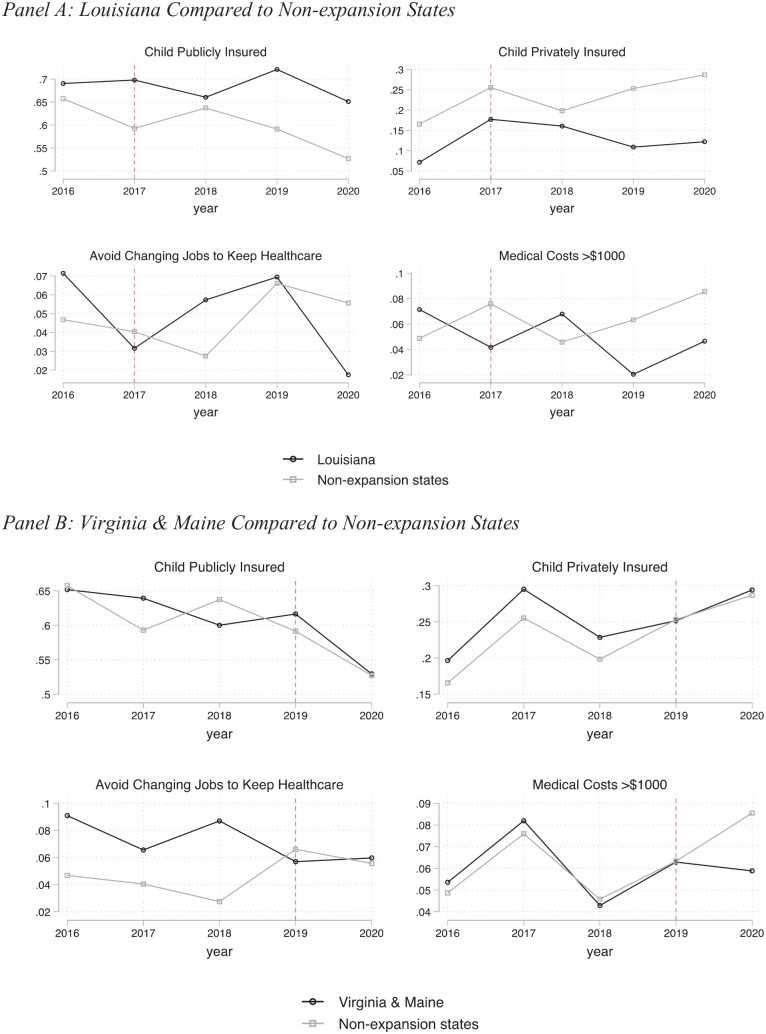
Raw trends in outcomes: Panel A: Louisiana compared to non-expansion States and Panel B: Virginia and Maine compared to non-expansion States.

Table 1 reports the means across treatment and comparison states, before and after expansion (years 2019-2020 for non-expansion states). The DD approach we employ relies on changes in trends across these 2 groups to isolate the effects of the policy; therefore, the differences in levels across these groups of states are not problematic per se. In Figure 2, we visually present the raw trends in the primary outcomes of interest between 2016 and 2020 comparing expansion states by date to non-expansions.^i^ These trends are approximately similar.

### Effects of the Expansions on Child Insurance Coverage, Parents’ Employment Decisions, and Family Health-Related Financial Well-being

We present the main results in Table 2 with each column of each panel corresponding to a different regression, as specified in equation (1). We report the coefficients of interest ( $\beta_{1}$ ), the cluster robust p-values, wild bootstrap p-values, wild bootstrap 95% confidence interval, means of the dependent variables, relative percentage change of the estimated effect with respect to the mean, and the number of observations. Panel A displays the results for children’s health insurance coverage. The DD estimate is positive and statistically significant for children’s public health insurance coverage implying an increase of about 5.6 percentage points in public health insurance among children as a result of the ACA Medicaid expansions for low-income parents (*P* < .01 under the standard approach to clustering standard errors; *P* < .10 under the more conservative wild cluster bootstrap).^j^ Relative to the mean of 66.2%, this estimate represents an increase of about 8.5%.

**Table 2.** Full Analytical Sample of Children.

	Panel A: Child insurance coverage			
	Child publicly insured	Child privately insured	Child both privately and publicly insured	Child uninsured
				
Expansion	**0.0560**	**−0.0494**	**−**0.0145	0.0079
Cluster robust *P*-value	**(.003)**	**(.022)**	(.505)	(.636)
Wild bootstrap*P*-value	**.0831**	**.0751**	.7317	.6356
Wild bootstrap 95% CI	**[−0.0622, .0997]**	**[−0.2681, .0615]**	[**−**0.1591, 0.1128]	[**−**0.0324, 0.1758]
Mean Y in expansion states in 2016	**0.662**	**0.162**	0.091	0.084
Relative % change	**8.5**	**−30.4**	**−**15.9	9.4
N	**4382**	**4382**	4382	4382
*R* ^2^	**0.156**	**0.250**	0.012	0.047

	Panel B: Medical-related financial well-being	
	Avoid Changing Jobs	Child’s medical costs exceed $1000
Expansion	**−0.0466**	**−0.0415**
Cluster robust *P*-value	**(.000)**	**(.012)**
Wild bootstrap *P*-value	**.0711**	**.032**
Wild bootstrap 95% CI	**[−0.1102, 0.0408]**	**[−0.3020, −0.0098]**
Mean Y in expansion states in 2016	**0.086**	**0.058**
Relative % change	**−54.5**	**−71.0**
N	**4319**	**4382**
*R* ^2^	**0.018**	**0.058**

*Source*. National Survey of Children’s Health, 2016 to 2020^47^; Kaiser Family Foundation. Sample consists of children in households eligible for Medicaid according to state income thresholds.

Notes. *P*-values in parentheses, significance stars omitted due to differences in analytical standards. Wild bootstrap *P*-values < .10 are bolded. Expansion = 1 if child’s state expanded Medicaid between 2016 and 2019, and the survey year = post expansion. Expansion = 0 if child’s state never expanded, pre-expansion for expansion states, and for 2020 expansion states (Utah and Idaho). Early, on-time, and other late expanders that expanded before 2016 were dropped. Regressions adjust for child’s sex, race/ethnicity, and age, whether child is less than 6 months of age, whether there are 3 or more children in the household, mother’s marital status, education level, and employment status, and whether the household speaks English as first language, state unemployment rates, state Earned Income Tax Credit (EITC) rates, whether EITC is refundable, state minimum wages, and the maximum Temporary Assistance for Needy Family (TANF) and Supplemental Nutrition Assistance Program (SNAP) combined benefits for a family of 3. 99% of publicly insured children are covered by Medicaid or CHIP.

Next, we observe a similarly sized absolute decrease in children’s private health insurance coverage: 4.9 percentage points. Because of the smaller base, however, this relative change is much larger: about 30% (*P* < .10). This finding suggests a crowd-out of private health insurance by public insurance. We do not observe any statistically significant effect of parental Medicaid expansions on dual insurance coverage (both private and publicly insured children) or on uninsurance.

Panel B shows the results for the medical-related financial well-being measures (job lock and high medical expenses). We find that parents are 4.6 percentage points (or about 55%) less likely to avoid job changes due to worries about losing employer-provided health insurance for their child (*P* < .10). Finally, the estimate is negative and statistically significant for children’s out-of-pocket medical costs exceeding $1000 a year implying decreases in the likelihood of families experiencing very high healthcare costs related to children. Specifically, we observe a 4-percentage point decrease in the likelihood of incurring medical costs for the focal child that exceeds $1000, which represents an approximately 71% decrease relative to the mean of 6% (*P* < .10). This large effect may suggest that the largest financial benefits of the expansions are concentrated among families with the greatest healthcare expenses.^k^

### Sensitivity Analyses

We conduct a falsification test on a group of children unexpected to be affected by the ACA’s Medicaid expansions that targeted low-income families. Specifically, we estimate our baseline model specification (1) on children from households with reported income equal to or greater than 400% FPL. As expected, Table 3 shows no significant change in children’s public insurance coverage or other health-related financial wellbeing outcomes for this group. The magnitude of the coefficients is also close to 0 for all outcomes, which bolsters confidence in the baseline results. Our results are also robust to alternative sample construction and model specifications (see Appendix D for Supplemental analyses).

**Table 3.** Falsification Test: High-Income Sample of Children (≥400 FPL).

	Panel A: Child insurance coverage			
	Child publicly insured	Child privately insured	Child both privately and publicly insured	Child uninsured
Expansion	0.0056	**−**0.0082	0.0008	0.0018
Cluster robust *P*-value	(.304)	(.241)	(.832)	(.642)
Wild bootstrap *P*-value	.344	.272	.807	.741
Wild bootstrap 95% CI	[**−**0.0545, 0.0416]	[**−**0.0498, 0.0444]	[**−**0.0444, 0.0338]	[**−**0.0403, 0.0336]
Mean Y in expansion states in 2016	0.031	0.942	0.009	0.017
Relative % change	17.8	**−**0.9	8.8	10.4
N	20 663	20 663	20 663	20 663
*R* ^2^	0.094	0.085	0.013	0.012

	Panel B: Medical-related financial well-being	
	Avoid Changing Jobs	Child’s medical costs exceed $1000
Expansion	−0.0061	−0.0093
Cluster robust *P*-value	(.461)	(.577)
Wild bootstrap *P*-value	.5265	.8378
Wild bootstrap 95% CI	[−0.1106, 0.0131]	[−0.076, 0.0920]
Mean *Y* in Expansion States in 2016	0.046	0.224
Relative % Change	−13.1	−4.2
N	20 705	20 687
*R* ^2^	0.006	0.015

*Source*. National Survey of Children’s Health, 2016-2020^47^; Kaiser Family Foundation. Sample consists of children in households with incomes > 400% FPL.

*Note. P*-values in parentheses, significance stars omitted due to differences in analytical standards. Wild bootstrap *P*-values < .10 are bolded. Expansion = 1 if child’s state expanded Medicaid between 2016 and 2019, and the survey year = post expansion. Expansion = 0 if child’s state never expanded, pre-expansion for expansion states, and for 2020 expansion states (Utah and Idaho). Early, on-time, and other late expanders that expanded before 2016 were dropped. Regressions adjust for child’s sex, race/ethnicity, and age, whether child is less than 6 months of age, whether there are 3 or more children in the household, mother’s marital status, education level, and employment status, and whether the household speaks English as first language, state unemployment rates, state Earned Income Tax Credit (EITC) rates, whether EITC is refundable, state minimum wages, and the maximum Temporary Assistance for Needy Family (TANF) and Supplemental Nutrition Assistance Program (SNAP) combined benefits for a family of 3. 99% of publicly insured children are covered by Medicaid or CHIP.

## Discussion

In this paper, we examine the short-term effects of expanding public health insurance eligibility for low-income adults on children’s public health insurance coverage, parents’ employment decisions, and low-income households’ health-related financial well-being as it relates to their children. We use data on children from low-income families to isolate a targeted sample of children whose parents were eligible for Medicaid through the ACA’s Medicaid expansions beyond 2015. These data provide detailed measures of child health insurance coverage and health-related financial well-being, enabling us to examine several specific mechanisms highlighted by theory and prior research.^26,61-63^ We contribute to the existing literature, which has primarily focused on “childless adults,” to understand the implications of the ACA Medicaid expansions on the health-related financial well-being of families with children.

First, we found that the ACA Medicaid expansions for low-income parents between 2016 and 2019 were associated with increases children’s public health insurance coverage by 5.6 percentage points (about 8.5%). Our findings are consistent with past literature on the welcome mat effects of public health insurance expansions for adults for already eligible children.^24,64^ Specifically, to put this in context with prior work, using data from the American Community Survey, Hudson and Moriya^24^ estimate a welcome mat effect of 5.7 percentage points (8.1%) for children as a result of the ACA expansions to adults.^l^ In contrast to Hudson and Moriya & Moriya,^24^ however, we find stronger evidence of public health insurance crowding out private insurance; children’s private insurance coverage declined by 4.9 percentage points (30%). These results call for an ongoing monitoring and research of public and private coverage options among low-income families and their associated trade-offs as suggested by prior literature.^65^ Findings imply that policies that expand parents’ public health insurance eligibility can have a significant impact on children’s health insurance coverage, and that these effects extend to late-expansion states.

Second, we find that the expansions lowered the financial burden of low-income families by reducing parental job lock and out-of-pocket medical costs for children’s healthcare. Specifically, the ACA Medicaid expansions for low-income parents reduced the likelihood that parents avoid changing jobs for reasons related to health insurance by about 4.5 percentage points (roughly 55%). This finding is consistent with Hamersma and Kim^44^ who examine earlier Medicaid expansions for parents. It differs, however, from other work using ACA insurance policies. For example, Callison and Sicilian and Sicilian^38^ find mixed effects of the expansions on labor market outcomes based on race/ethnicity and gender. When examining the ACA’s young adult mandate, Bailey and Chorniy^40^ find no effects on job lock while Kofoed and Frasier^39^ show reductions in job lock among enlisted soldiers in the U.S. Army. These differences suggest that parents are more sensitive to changes in health insurance eligibility when it comes to job mobility.

Finally, these expansions reduced the likelihood of these families incurring high ($1000 or more per year) child medical costs by 4 percentage points. Health-related financial burdens are notable among low-income families.^1,3^ Previous research has documented that the ACA expansions reduced healthcare related financial strain,^10^ yet this research was not specific to parents nor to child medical costs. Additional research focusing on households with children found the ACA reduced healthcare-related financial burden,^30^ but was unable to separate the role of Medicaid expansions from other health insurance provisions (e.g., the ACA marketplace) or child medical expenses from other household members. Our findings suggest that there were financial benefits for low-income families in late-expansion states through reductions in high child medical bills, and that these financial burdens are lowered primarily for high medical expense households. The results suggest that policies that expand low-income parents’ public health insurance eligibility may reduce parents’ high child medical expenses, especially among families with higher expenses.

## Limitations

We use a quasi-experimental design to estimate the effect of adult Medicaid eligibility rather than a random assignment experimental design. There may be omitted, time-varying variables that are correlated with both our financial well-being outcomes and whether a state expanded their Medicaid program. We use strategies to reduce this concern, including the use of a control group of states, state and wave fixed effects, and adjusting for child- and family-level covariates and state-level time-varying characteristics. We also estimate several alternative sample construction and model specifications (including wild cluster bootstrap methods), and multiple placebo checks.

Similarly, robust, closed-form, power formulas for observational studies, such as the quasi-experimental DD analysis we conduct in this study, are not readily available.^66^ We conducted a post-hoc power calculation using emerging guidance^67^ and find that we are sufficiently powered to estimate minimum detectable effect size (MDE) in the ballpark of what prior literature shows.^m^

Finally, all measures used in this study are self-reported. As an example, respondents may misreport public health insurance coverage as private. In this case, we also examined dual enrollment and uninsurance, but future research should examine more objective measures of families’ health-related financial well-being. Lastly, we have between 1 and 3 post-expansion waves in the dataset, varying by the state. Therefore, our results reflect only short-term effects. Further research should examine the long-term effects of the ACA Medicaid expansions on parents and their families.

## Conclusions and Implications

Our findings indicate that the ACA Medicaid expansions had important positive benefits for parents and children. Households experienced less parental job lock due to child health reasons and lower medical financial burden, particularly among high medical expense children. Our study adds to the accumulating evidence of the role of public health insurance expansions on health-related financial well-being,^3,6^ and highlights the “missed opportunities”^26^ for low-income parents in non-expansion states.

Furthermore, recent research has shown that many states that expanded Medicaid under the ACA were able to do so without substantial increases in administrative spending.^68^ Indeed, studies are uncovering other benefits for hospitals in states that are yet to expand, which include potential savings from lower costs of treatment and reduced costs of uncompensated care attributed to uninsured patients.^69^

Finally, research has documented the long-run benefits of health insurance coverage^70-73^ and household financial well-being^74^ for children’s health and developmental trajectories. This study suggests that it might be better late than never when it comes to states adopting the ACA Medicaid expansions, given that positive benefits might continue to accrue into the future.

## Figures and Tables

**Appendix D1.**
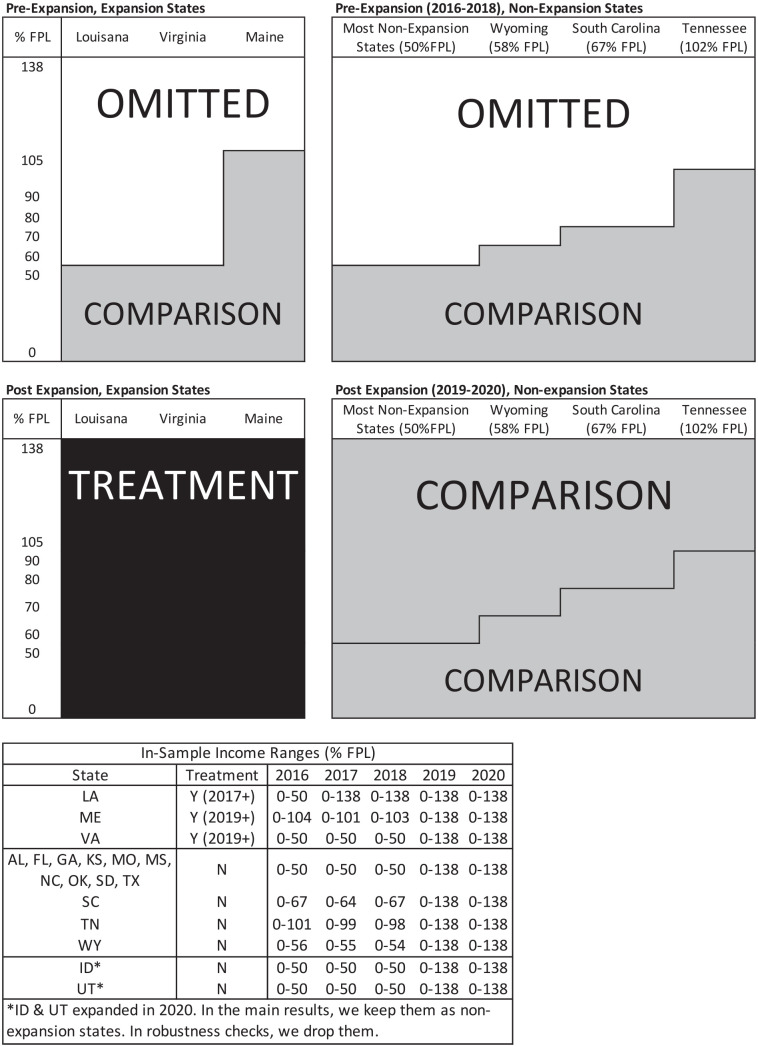
Conceptual Map of Treatment and Comparison Groups, Alternate Sample.

## Appendix A: Description of Covariates in Analysis

### Child and Family Characteristics

We adjust for several child- and family-level covariates; specifically, indicators for child sex, race/ethnicity, and age, whether child is less than 6 months of age, whether there are 3 or more children in the household, whether the household speaks English as first language, and mother’s marital status, education level, and employment status. Existing literature has linked these characteristics of children and parents with child underinsurance,^53^ out of pocket health care expenditures,^49^ difficulty in paying medical bills,^75^ and parents’ employment changes for health insurance reasons.^44^ Descriptive statistics of child and family characteristics in the analytic sample are shown in Table 1.

### State Characteristics

There are several state-level characteristics that may differentially affect the key outcome measures in households across states. Because these may also be correlated with state decisions to expand Medicaid, we adjust for these factors in our models. Specifically, we include the following characteristics: state unemployment rates, state Earned Income Tax Credit (EITC) rates, whether EITC is refundable, state minimum wages, and the maximum Temporary Assistance for Needy Family (TANF) and Supplemental Nutrition Assistance Program (SNAP) combined benefits for a family of 3.^n^ Each of these are measured at the state-year level.

For more in-depth information regarding the data including codebooks, questionnaire, and measures, please refer to publicly available documentation from NSCH here: https://www.childhealthdata.org/learn-about-the-nsch/NSCH.

**Appendix B.** Timing of ACA Medicaid Late Expansion States.

	Expansion states	Date of expansion	Effective year coded in this Study	Non-expansion states
1	Louisiana	July 2016	2017	Alabama
2	Virginia	January 2019	2019	Florida
3	Maine	January 2019	2019	Georgia
4				Idaho*
5				Kansas
6				Mississippi
7				Missouri
8				Nebraska
9				North Carolina
10				Oklahoma
11				South Carolina
12				Tennessee
13				Texas
14				Utah*
15				Wyoming

* Idaho and Utah expanded in 2020. Sensitivity checks in Appendix D drop these states from the analysis.

**Appendix C.** Results Among Sample of Children with Chronic Conditions.

	Panel A: Child insurance coverage			
	Child publicly insured	Child privately insured	Child both privately and publicly insured	Child uninsured
Expansion	0.0360	**−0.0832**	0.0073	**0.0399**
Cluster robust *P*-Value	(.120)	**(.053)**	(.848)	**(.060)**
Wild bootstrap *P*-Value	.1612	**.0801**	.8328	**.0791**
Wild bootstrap 95% CI	[**−**0.0311, 0.0794]	**[−0.2920, 0.4251]**	[−0.0659, 0.1272]	**[−0.0094, 0.2424]**
Mean Y in expansion states in 2016	0.742	**0.161**	0.032	**0.064**
Relative % Change	4.9	**−51.6**	22.6	**61.8**
N	1484	**1484**	1484	**1484**
*R* ^2^	0.127	**0.217**	0.024	**0.064**

	Panel B: Medical–related financial well–being	
	Avoid Changing Jobs	Child’s medical costs exceed $1000
Expansion	**−0.0756**	**−0.0737**
Cluster robust *P*-Value	**(.016)**	**(.000)**
Wild bootstrap *P*-Value	**.042**	**.0631**
Wild bootstrap 95% CI	**[−0.1781, −0.0156]**	**[−0.2452, 0.0439]**
Mean Y in expansion states in 2016	**0.148**	**0.081**
Relative % change	**−51.2%**	**−91.4%**
N	**1450**	**1484**
*R* ^2^	**0.036**	**0.089**

*Source*. National Survey of Children’s Health, 2016 to 2020^47^; Kaiser Family Foundation. Sample consists of children in households eligible for Medicaid according to state income thresholds who had been diagnosed with a chronic condition at the time of the survey.

*Notes. P*-values in parentheses, significance stars omitted due to differences in analytical standards. Wild bootstrap *P*-values < .10 are bolded. Expansion = 1 if child’s state expanded Medicaid between 2016 and 2019, and the survey year = post expansion. Expansion = 0 if child’s state never expanded, pre-expansion for expansion states, and for 2020 expansion states (Utah and Idaho). Early, on-time, and other late expanders that expanded before 2016 were dropped. Regressions adjust for child’s sex, race/ethnicity, and age, whether child is less than 6 months of age, whether there are 3 or more children in the household, mother’s marital status, education level, and employment status, and whether the household speaks English as first language, state unemployment rates, state Earned Income Tax Credit (EITC) rates, whether EITC is refundable, state minimum wages, and the maximum Temporary Assistance for Needy Family (TANF) and Supplemental Nutrition Assistance Program (SNAP) combined benefits for a family of 3. 99% of publicly insured children are covered by Medicaid or CHIP.

## Appendix D. Supplementary Analyses

The main analytical sample consists of only children in households whose parents were eligible for Medicaid based on their state’s income thresholds. Though inclusion in the sample is based upon state Medicaid policy decisions (rather than individual characteristics), one potential concern in using this approach is if any observable effects are driven by changes in sample composition across time and within state. In other words, we want to be certain that any observed effects are only because of the change in the eligibility threshold (% FPL) in the expansion states at the time of the expansion. Therefore, we perform a robustness check where we include in the sample children in households with incomes between state-specific thresholds and 138% FPL in control states in 2019 and 2020 (i.e., households between 50 and 138 in most control states). This exercise tests whether including slightly higher income households in the treatment states post expansion is driving the effects. Appendix D1 shows a conceptual figure of this sample construction. Appendix D2 erports the descriptive statistics for this alternate sample, and Appendix D3 reports the results. The results are slightly attenuated, though substantively the same. These findings offer evidence that the observed effects are not due to differences in people at the newly eligible thresholds, but rather due to the state-specific policy changes.

Next, Utah and Idaho both expanded their Medicaid programs to 138% FPL with enrollment beginning in January 2020. In the main results, we treat them as non-expansion states since some outcomes inquire about the past 12 months and effects would likely be attenuated for the questions asking about current status (e.g., child health insurance coverage). In a robustness check, we drop these 2 states entirely. As seen in Appendix D4, this decision is not consequential for the results. The coefficient estimates are nearly identical, though slightly less precise. The reduction in precision is likely due to dropping 2 out of 18 clusters in the wild cluster bootstrap.

Finally, to examine if a single expansion state has a stronger influence on the average effects, we perform a “leave one out” analysis. Specifically, we drop observations from each expansion state from the analysis sequentially to determine the sensitivity of the results. These results are in Appendix D5. Due to limited statistical power, the effects are imprecise. However, the point estimates are consistent across all models providing suggestive evidence that the benefits were widely shared in each of these late-expansion states.

**Appendix D2.** Descriptive Statistics for Alternate Sample.

	Expansion state (Louisiana, Maine, Virginia)		Non-expansion states	
	Pre-expansion	Post-expansion	2016-2018	2019-2020
Outcomes				
Child publicly insured	0.64	0.64	0.63	0.58
Child privately insured	0.21	0.19	0.20	0.25
Child publicly and privately insured	0.06	0.07	0.05	0.06
Child uninsured	0.09	0.10	0.11	0.10
Avoided changing jobs due to concerns about maintaining health insurance for child	0.08	0.05	0.04	0.06
Child medical costs exceed $1000	0.06	0.05	0.06	0.07
Household/child variables				
Household speaks English (Primary Language)	0.92	0.92	0.87	0.88
Non-Hispanic White	0.73	0.57	0.59	0.65
Non-Hispanic Black	0.15	0.32	0.25	0.21
Other race	0.13	0.11	0.16	0.13
Hispanic	0.06	0.09	0.18	0.19
Mother is married	0.44	0.39	0.38	0.44
Mother education ≤ High school	0.07	0.09	0.13	0.10
Mother education = High school Grad	0.29	0.30	0.28	0.27
Mother education ≥ High school	0.49	0.44	0.42	0.46
Mother employed	0.36	0.47	0.37	0.49
Three or More children in household	0.31	0.27	0.29	0.29
Child is male	0.50	0.50	0.53	0.53
Child’s age (years)	8.7	9.3	8.8	9.3
Child is under 6 months	0.01	0.01	0.02	0.02
Household income (% FPL)	61.5	80.5	52.6	83.3
State variables				
Unemployment rate	0.04	0.05	0.04	0.05
State minimum wage ($)	8.03	8.16	7.39	7.46
State EITC rate	0.09	0.08	0.02	0.05
EITC refundable	0.33	0.81	0.09	0.05
Maximum TANF/SNAP benefit for family of 3 ($)	955	860	807	829
N	285	940	1690	3727

*Source.* NSCH 2016 to 2020.

*Note*. Sample consists of households with incomes at or below their state’s parental eligibility thresholds for 2016 to 2018, and <138 FPL for 2019 to 2020. Descriptive statistics are not weighted due to limits on the analytic sample.

**Appendix D3.** Alternate Sample, <138 FPL for 2019 to 2020.

	Panel A: Child insurance coverage			
	Child publicly insured	Child privately insured	Child both privately and publicly insured	Child uninsured
Expansion	**0.0378**	**−0.0358**	−0.0161	0.0142
Cluster robust *P*-value	**(.019)**	**(.108)**	(.434)	(.432)
Wild bootstrap *P*-value	**.0831**	**.0771**	.7087	.4725
Wild bootstrap 95% CI	**[−0.0220, 0.1293]**	**[−0.3624, 0.0077]**	[−0.1665, 0.1401]	[**−**0.0295, 0.2161]
Mean Y in Expansion States in 2016	**0.662**	**0.162**	0.091	0.084
Relative % Change	**5.7**	**−22.1**	−17.7	16.8
N	**6642**	**6642**	6642	6642
R2	**0.142**	**0.211**	0.009	0.040

	Panel B: Medical-related financial well-being	
	Avoid changing jobs	Child’s medical costs exceed $1000
Expansion	**−0.0411**	**−**0.0248
Cluster robust *P*-value	**(.000)**	(.061)
Wild bootstrap *P*-value	**.0951**	.1251
Wild bootstrap 95% CI	**[−0.1298, 0.0499]**	[**−**0.1945, 0.0313]
Mean Y in expansion states in 2016	**0.086**	0.058
Relative % change	**−48.1**	**−**42.4
N	**6558**	6642
*R* ^2^	**0.010**	0.047

*Source*. National Survey of Children’s Health, 2016 to 2020^47^; Kaiser Family Foundation. Sample consists of children in households eligible for Medicaid according to state income thresholds for 2016 to 2018, and up to 138 FPL for all states in 2019 to 2020.

*Note. P*-values in parentheses, significance stars omitted due to differences in analytical standards. Wild bootstrap *P*-values < .10 are bolded. Expansion = 1 if child’s state expanded Medicaid between 2016 and 2019, and the survey year = post expansion. Expansion = 0 if child’s state never expanded, pre-expansion for expansion states, and for 2020 expansion states (Utah and Idaho). Early, on-time, and other late expanders that expanded before 2016 were dropped. Regressions adjust for child’s sex, race/ethnicity, and age, whether child is less than 6 months of age, whether there are 3 or more children in the household, mother’s marital status, education level, and employment status, and whether the household speaks English as first language, state unemployment rates, state Earned Income Tax Credit (EITC) rates, whether EITC is refundable, state minimum wages, and the maximum Temporary Assistance for Needy Family (TANF) and Supplemental Nutrition Assistance Program (SNAP) combined benefits for a family of 3. 99% of publicly insured children are covered by Medicaid or CHIP.

**Appendix D4.** Full Analytical Sample of Children, Dropping Idaho and Utah.

	Panel A: Child insurance coverage			
	Child publicly Insured	Child privately insured	Child both privately and publicly insured	Child uninsured
Expansion	0.0449	**−0.0411**	**−**0.0153	0.0114
Cluster robust *P*-value	(.007)	**(.052)**	(.486)	(.526)
Wild bootstrap *P*-value	.1011	**.0941**	.6907	.5205
Wild bootstrap 95% CI	[**−**0.1015, 0.0801]	**[−0.292, 0.0374]**	[**−**0.1211, 0.0661]	[**−**0.0343, 0.1594]
Mean Y in expansion states in 2016	0.662	**0.162**	0.091	0.084
Relative % Change	6.8	**−25.3**	−16.8	13.5
N	3969	**3969**	3969	3969
R2	0.142	**0.254**	0.013	0.041

	Panel B: Medical-related financial well-being	
	Avoid Changing Jobs	Child’s medical costs exceed $1000
Expansion	**−0.0457**	**−0.0416**
Cluster robust *P*-Value	**(.000)**	**(.014)**
Wild bootstrap *P*-Value	**.0611**	**.023**
Wild bootstrap 95% CI	**[−0.1012, .0193]**	**[−0.2927, −0.0135]**
Mean *Y* in expansion States in 2016	**0.086**	**0.058**
Relative % change	**−53.4%**	**−71.2%**
N	**3914**	**3969**
*R* ^2^	**0.018**	**0.057**

*Source*. National Survey of Children’s Health, 2016 to 2020^47^; Kaiser Family Foundation. Sample consists of children in households eligible for Medicaid according to state income thresholds.

*Notes. P*-values in parentheses, significance stars omitted due to differences in analytical standards. Wild bootstrap *P*-values < .10 are bolded. Expansion = 1 if child’s state expanded Medicaid between 2016 and 2019, and the survey year = post expansion. Expansion = 0 if child’s state never expanded, pre-expansion for expansion states. Early, on-time, and other late expanders that expanded before 2016 were dropped. Regressions adjust for child’s sex, race/ethnicity, and age, whether child is less than 6 months of age, whether there are 3 or more children in the household, mother’s marital status, education level, and employment status, and whether the household speaks English as first language, state unemployment rates, state Earned Income Tax Credit (EITC) rates, whether EITC is refundable, state minimum wages, and the maximum Temporary Assistance for Needy Family (TANF) and Supplemental Nutrition Assistance Program (SNAP) combined benefits for a family of 3. 99% of publicly insured children are covered by Medicaid or CHIP.

**Appendix D5.** Leave One Out Analysis.

	Child publicly insured	Child privately insured	Child both privately and publicly insured	Child uninsured	Avoid changing jobs	Child’s medical costs exceed $1000
Panel A: Main results						
Expansion	**0.0560**	**−0.0494**	**−**0.0145	0.0079	**−0.0466**	**−0.0415**
Cluster robust *P*-value	**(.003)**	**(.022)**	(.505)	(.636)	**(.000)**	**(.012)**
Wild bootstrap *P*-value	**.0831**	**.0751**	.7317	.6356	**.0711**	**.032**
N	**4382**	**4382**	4382	4382	**4319**	**4382**
Panel B: Leave out Virginia						
Expansion	0.0498	**−**0.0464	**−**0.0241	0.0207	**−**0.0487	**−0.0582**
Cluster robust *P*-value	(.048)	(.077)	(.412)	(.289)	(.008)	**(.018)**
Wild bootstrap *P*-value	.2513	.1471	.8128	.3804	.2533	**.0861**
N	4118	4118	4118	4118	4056	**4118**
Panel C: Leave out Maine						
Expansion	**0.0587**	**−**0.0470	**−**0.0265	0.0148	**−**0.0366	**−0.0378**
Cluster robust *P*-value	**(.001)**	(.042)	(.303)	(.322)	(.003)	**(.002)**
Wild bootstrap *P*-value	**.0801**	.2122	.5606	.2923	.1522	**.0921**
N	**4040**	4040	4040	4040	3983	**4040**
Panel D: Leave out Louisiana						
Expansion	0.0592	**−**0.0701	0.0115	**−**0.0006	**−**0.0586	**−0.0484**
Cluster robust *P*-value	(.042)	(.005)	(.470)	(.977)	(.000)	**(.043)**
Wild bootstrap *P*-value	.3243	.1582	.4735	.984	.1041	**.0911**
N	3763	3763	3763	3763	3710	**3763**

*Source*. National Survey of Children’s Health, 2016 to 2020^47^; Kaiser Family Foundation. Sample consists of children in households eligible for Medicaid according to state income thresholds.

*Note. P*-values in parentheses, significance stars omitted due to differences in analytical standards. Wild bootstrap *P*-values < .10 are bolded. Expansion = 1 if child’s state expanded Medicaid between 2016 and 2019, and the survey year = post expansion. Expansion = 0 if child’s state never expanded, pre-expansion for expansion states, and for 2020 expansion states (Utah and Idaho). Early, on-time, and other late expanders that expanded before 2016 were dropped. Each panel represents dropping one of the treatment states. Regressions adjust for child’s sex, race/ethnicity, and age, whether child is less than 6 months of age, whether there are 3 or more children in the household, mother’s marital status, education level, and employment status, and whether the household speaks English as first language, state unemployment rates, state Earned Income Tax Credit (EITC) rates, whether EITC is refundable, state minimum wages, and the maximum Temporary Assistance for Needy Family (TANF) and Supplemental Nutrition Assistance Program (SNAP) combined benefits for a family of 3. 99% of publicly insured children are covered by Medicaid or CHIP.

**Appendix E.** Full Analytical Sample of Children, Probit Models.

	Panel A: Child insurance coverage			
	Child publicly insured	Child privately insured	Child both privately and publicly insured	Child uninsured
Expansion	0.1414	−0.1922	−0.0990	0.0269
Cluster robust *P*-value	(.003)	(.104)	(.482)	(.791)
Wild bootstrap *P*-value	.1491	.2232	.6747	.7968
N	4382	4382	4382	4382

	Panel B: Medical-Related Financial Well-being	
	Avoid changing jobs	Child’s medical costs exceed $1000
Expansion	**−0.4550**	**−0.3364**
Cluster robust *P*-value	**(.000)**	**(.008)**
Score bootstrap *P*-value	**.019**	**.02**
N	**4319**	**4382**

*Source*. National Survey of Children’s Health, 2016 to 2020^47^; Kaiser Family Foundation. Sample consists of children in households eligible for Medicaid according to state income thresholds.

*Note. P*-values in parentheses, significance stars omitted due to differences in analytical standards. Score bootstrap *P*-values < .10 are bolded. Expansion = 1 if child’s state expanded Medicaid between 2016 and 2019, and the survey year = post expansion. Expansion = 0 if child’s state never expanded, pre-expansion for expansion states, and for 2020 expansion states (Utah and Idaho). Early, on-time, and other late expanders that expanded before 2016 were dropped. Regressions adjust for child’s sex, race/ethnicity, and age, whether child is less than 6 months of age, whether there are 3 or more children in the household, mother’s marital status, education level, and employment status, and whether the household speaks English as first language, state unemployment rates, state Earned Income Tax Credit (EITC) rates, whether EITC is refundable, state minimum wages, and the maximum Temporary Assistance for Needy Family (TANF) and Supplemental Nutrition Assistance Program (SNAP) combined benefits for a family of 3. 99% of publicly insured children are covered by Medicaid or CHIP.

## Notes

a. The previous wave of NSCH was in 2012; however, the survey was redesigned in 2016 and therefore prior waves are not comparable to the 2016 to 2020 waves we use in our analytical sample. This also necessitated that we focus on children and families residing in states that expanded in 2016 or later. Furthermore, there is reason to believe that these late expansions may also have differential effects when compared to the first wave of ACA expansions. For example, Courtemanche and colleagues^76^ finds insurance coverage gains falling between 2016 and 2019.

b. Specifically, the sample consists of the following expansion states: Louisiana, Maine, Virginia, and non-expansion states: Alabama, Florida, Georgia, Idaho, Kansas, Missouri, Mississippi, North Carolina, Oklahoma, South Carolina, South Dakota, Tennessee, Texas, Utah, and Wyoming. Utah and Idaho both expanded in 2020. Due to the question wording for some questions asking about the past 12 months, we treat Utah and Idaho as non-expansion states in the main results. In robustness checks, we drop these 2 states from the analysis and the results are the same (Table A1).

c. See https://www.kff.org/medicaid/state-indicator/medicaid-income-eligibility-limits-for-parents/for more details on income thresholds for parents that we use.

d. Public insurance includes any type of government assistance. 99% of publicly insured children are covered by Medicaid or CHIP. Private insurance includes privately purchased, including through ACA marketplace, through employer, or TRICARE.

e. Medical costs include co-pays and amounts reimbursed from Health Savings Accounts (HSA) and Flexible Spending Accounts (FSA). It does not include health insurance premiums or costs that were or will be reimbursed by insurance or another source as previously used in the literature30.

f. The results are substantively similar, though smaller in magnitude when using a $500 cutoff. These modest differences suggests stronger effects for children with the highest medical expenses.

g. The NSCH bottom codes income as a percent of FPL at 50. Because some of these households would be considered newly eligible in states with pre-ACA parental eligibility thresholds less than 50% FPL, and there is evidence of a welcome mat effect among the previously eligible (e.g., McMorrow and Kenney29), we consider all households with incomes <50% in these states to be in the treatment group, as shown in Figure 1.

h. Results are substantively the same when estimating a non-linear model. Appendix E shows results using a probit model.

i. Indeed, the main identification assumption is that, absent the ACA Medicaid expansions for low-income parents, outcomes for children and families in eligible households in expansion states and non-expansion states would have continued the same trends despite differing average levels. Because we do not have data from multiple years prior to the expansions, we cannot test directly for differential pre-trends in our sample as evidence that this assumption likely holds.

j. We only report the wild cluster bootstrap method going forward in the manuscript. The tables include both standard clustered errors as well as the wild bootstrap-based standard errors.

k. To probe this result further we limit the sample to children who had been diagnosed with a chronic condition at the time of the survey. Chronic conditions include asthma, cerebral palsy, diabetes, heart condition, headaches, epilepsy, Tourette syndrome, depression, behavior problems, developmental delay, intellectual disability, speech disorder, learning disability, autism ASD, and ADD/ADHD. Approximately 34% of the sample had been diagnosed with one of these chronic conditions. In Appendix C, we show that the financial well-being results are stronger among this sample. The effects on child health insurance coverage are attenuated, however, and less precise. These results suggest that the benefits of parental health insurance expansions on health-related financial wellbeing may not be equally shared across all children, even among those from low-income families. We are cautious in interpreting these results, however, due to the substantially smaller size of this sample.

l. Of note, the ACS is nationally representative of housing units. The NSCH is representative of the nation’s child population.

m. For a DD analysis using repeated cross-sectional data on about 50 children within each state, 3 treatment states and 15 comparison states, observed across 5 time-periods (given our sample of approximate 4500 child-year observations in our baseline specification), we estimate that we are powered to detect an MDE ranging between 0.24 and 0.35 (power ranging from 0.60 and 0.90, respectively) using the formulas provided by Schochet^67^.

n. The results are not sensitive to excluding these state-level covariates.
